# Status of drowning in Nepal: A study of central police data

**DOI:** 10.12688/f1000research.14563.2

**Published:** 2018-07-30

**Authors:** Bhagabati Sedain, Puspa Raj Pant

**Affiliations:** 1 Padma Kanya Multiple Campus, Tribhuvan University, Kathmandu, Nepal; 2 Centre for Child and Adolescent Health, University of the West of England, Bristol, UK

**Keywords:** Drowning, Nepal, Public Health, Natural water bodies, Injury Prevention

## Abstract

**Background**:  Drowning is a serious and mostly preventable injury-related cause of death. Low-and-middle income countries represent over 90% of total drowning deaths worldwide. There is lack of epidemiological studies of drowning in Nepal. The aim of this paper is to describe the status of drowning in Nepal.

**Methods**: Cases of drowning, occurring between 1 January 2013 and 31 December 2015 were extracted from the Daily Incident Recording System of Nepal Police. Drowning cases were extracted and analysed regardless of their intent. Variables on age, sex of the deceased, types of water bodies, geographical locations, season when drowning occurred and activities of deceased were extracted and descriptive analysis was conducted.

**Results**: A total of 1,507 drowning cases were recorded over a 3 year period. The rate of drowning was 1.9 per 100,000 (2.95 for males and 0.92 for females). Majority of drowning occurred among males (76%) and more than half were (53%) under 20 years of age. Mostly drowning occurred in rivers (natural water bodies). The findings provide strong indication that drowning occurs throughout the year in Nepal. Children were highly vulnerable to drowning. The magnitude of drowning was found to be lower than estimated by global burden of disease (GBD) study.

**Conclusion**: The burden of drowning in Nepal is considerable, but mostly unknown to the public. Despite only having access to a limited data source, this study provides useful evidence that comprehensive research in Nepal is needed urgently.

## Introduction

Drowning is gradually being recognized as a leading cause of death in the low-and-middle-income countries (LMIC); yet it remains a neglected problem in many countries in the absence of adequate data ( Peden *et al*., 2008; Rahman *et al*., 2009). The World Health Organization (WHO) estimated 372,000 deaths every year occur due to drowning, making it the world’s third leading cause of fatal unintentional injuries ( WHO, 2014). About 91% of the drowning deaths occur in low- and middle- income countries ( Peden *et al*., 2008). Similarly, 40% of the world's total drowning deaths occur in children below 15 years and most of these occurred in low- and middle-income countries; 29% in South-East Asia region alone ( IHME, 2016). A study from Bangladesh found the fatal drowning rate to be as high as 15.8 per 100,000 ( Rahman *et al*., 2017).

**Figure 1.**
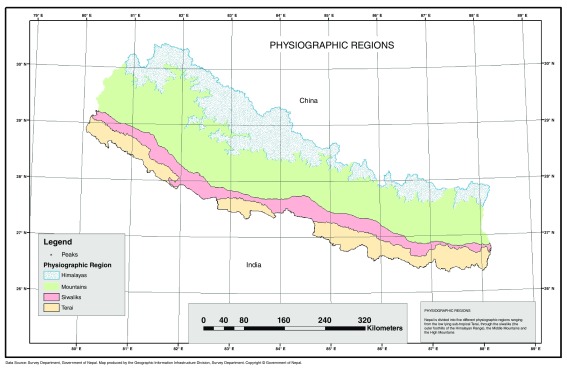
Map of Nepal indicating geographical regions. Reproduced with permission from the Survey Department, Ministry of Agriculture, Land Management and Cooperative.

A systematic review on epidemiology of drowning in LMICs has found that most studies on drowning were only from some countries in Asia: Bangladesh, China, India, Iran, Pakistan, Sri Lanka, Thailand and Vietnam ( Tyler *et al*., 2017). Nepal being a mountainous country; drowning does not come instantly to mind when considering the main causes of deaths. Nepal is divided into five different physiographic regions ranging from Sub-tropical Terai (plains) in the South to High Mountains covered with snow in the North ( Figure 1). These regions are often mentioned as three broad regions of Mountain, Hill and Terai in the administrative records. In Nepal, the summer monsoon season accounts for 80% of the annual rainfall, and the winter monsoon accounts for the remaining 20% ( DoHM, 2017). Flash floods, ditches, irrigation canals, and open wells are the chief hazards for drowning. Nepal has the poor coverage of vital event registration ( Gautam, 2016). According to the GBD estimates, an average of 1,300 people died in 2016 from drowning in Nepal with a mortality rate of 4.0 (95%UI 3.2 – 5.6) per 100,000 population ( IHME, 2016).

Detailed community-based studies on drowning have not been conducted in Nepal; however, there are some small-scale hospital-based post-mortem reports which present the cases of drowning. Drowning was the cause of death for 5% of total reported autopsy cases in a study of external causes of death reported in the Autopsy Centre in Kathmandu ( Lunetta *et al*., 2004; Sharma *et al*., 2006). Another hospital based study found drowning as a cause of death for 144 cases out of total 205 post-mortem cases in Kathmandu during 2005 to 2010. This study also found that 39% of the drowning victims were below 18 years of age and 24% below 11 years during 2005–2010 ( Wasti *et al*., 2010). However, these studies only included autopsy cases referred to hospital by the police as 'unnatural deaths,' which included deaths caused by drowning ( Sharma *et al*., 2006; Wasti *et al*., 2010).

Similarly, an analysis of media reports on drowning deaths conducted for April 2010 to April 2011 showed that over 200 cases of drowning deaths in Nepal were reported to media, with more than half of the total drowning deaths were children ( Pant *et al*., 2011). This study aims to discuss the status of drowning based on the officially recorded Nepal Police’s information on drowning deaths as a main data source.

## Methods

This study utilised drowning cases reported to Nepal Police during January 1 2013 to 31 December 2015. Family members or community people must report the incidents of drowning to the Daily Incidence Reporting Center of Nepal Police in each village development committee (VDC) level. Polices’ database records the facts and evidence pertinent to an incident.

Police records of drowning deaths is the only national source of drowning deaths recorded in Nepal. Drowning deaths are recorded in the form of event records (narrative) including the details of the place of the residence, age, date, sex, place of drowning, type of water body, intent (intentional or accidental drowning) and activity during drowning. Both the intentional and unintentional drowning cases were analysed in this study. The described variables included; location/ geographical region, water bodies, months of drowning were also extracted, entered and analysed using SPSS version 16.0.

Descriptive study was performed in order to show the distribution of fatal drowning by age, gender, type of water body, months of the year, geographical location, and activity of the victim before the drowning incident. Possible core victim identifiers i.e. age, gender, date, activity and location of the incident and describing variables i.e. water bodies, months of drowning, and activity before drowning were also analysed and described. Information about the 'time', 'distance' from the victim’s home and the companion were not available in the database.

## Ethics

Ethical approval was not required for this study, because it did not involve human participants. This study used secondary data registered by police. To access the data the request latter on behalf of Tribhuvan University, Padma Kanya Campus was submitted to the Crime Investigation Department. As per the request, the police department granted accesses to the police record. In this study, personal identifiers or confidential information of deceased were not disclosed.

## Results

This study identified 1,507 cases of drowning deaths in three years (2013–2015). Among these drowning cases highest deaths were in Terai (low land) 700 (46.4%). Drowning deaths were found to vary between geographical regions ( Table 1). Most of the drowning deaths in Nepal occurred in the natural water bodies. The highest number of drowning deaths were observed in the Plains followed by the Hills.

**Table 1.** Drowning deaths by geographical regions for the period 2013–2015.

Geographical region	Number (%)
Mountain	228 (15.1)
Hill	579 (38.4)
Terai	700 (46.4)
**Total**	**1507 (100.0)**

The proportion of males that died from drowning was higher than females. Table 2 shows the sex differentials in drowning deaths. Younger people (<20 years) comprised over half of the drowning deaths, making up 52.7% of all drowning deaths. Similarly, age specific drowning deaths were higher for 10–19 years closely followed by 0–9 years and then declined as the age increases. The most common drowning age was 10–19 years for males and 0–9 years for females. Age reporting was missing for 27 cases.

**Table 2.** Drowning deaths compared by age and sex for the period 2013–2015.

Age group (years)	Sex		Total
	Male Number (%)	Female Number (%)	
0–9	211 (18.8%)	117 (32.8)	328 (22.2%)
10–19	351 (31.3%)	100 (28.0%)	451 (30.5%)
20–29	196 (17.5%)	40 (11.2%)	236 (15.9%)
30–39	109 (9.7%)	40 (11.2%)	149 (10.1%)
40–49	100 (8.9%)	22 (6.2%)	122 (8.2%)
50–59	66 (5.9%)	18 (5.0%)	84 (5.7%)
60–69	53 (4.7%)	12 (3.4%)	65 (4.4%)
70–79	29 (2.6%)	5 (1.3%)	34 (2.3%)
80–89	8 (0.6%)	1 (0.3%)	9 (0.6%)
90–99	0 (0%)	2 (0.6%)	2 (0.1%)
**Total ^§^**	**1,123 (100%)**	**357 (100%)**	**1,480 (100%)**

§Age was not recorded for 27 cases

**Figure 2.**
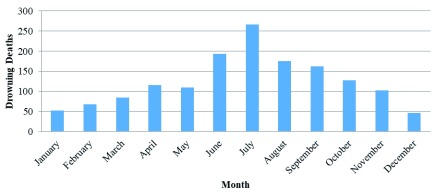
Percentage distribution of drowning cases by month (n-1507).

This study also identified a seasonal pattern of drowning, which showed high incidents of drowning during monsoon season, with peaks in July, with the winters (December–January) being relatively low for drowning. The monsoon season (June – August) claimed about 43% of the total drownings and over 15% drownings occurred in the summer season i.e. during April–May ( Figure 2).

More than 88% of the drowning deaths occurred in natural bodies of water. Rivers, ponds and lakes were most common places for drowning in Nepal ( Table 3). A smaller proportion of drowning also occurred in man-made water containing bodies like canals, water-filled pits, safety tanks, water tanks, and wells.

**Table 3.** Distribution by place of drowning for the period 2013–2015.

Source of drowning	Number (%)
River	1195 (79.3%)
Pond	114 (7.6%)
Canal	82 (5.4%)
Lake	19 (1.3%)
Water-filled pits	66 (4.4%)
Safety tank	5 (0.3%)
Water tank	7 (0.5%)
Wells	14 (0.9%)
Others	5 (0.3)
**Total**	**1507 (100%)**

Swimming, bathing, and crossing the river were the mostly reported activities before drowning, and occurring mainly in the summer season. In Nepal, whether people go near the river, bathe by the bank or go into the water for swimming depends upon age, gender and location of the river. Activity before drowning for 181 (12%) cases were not mentioned at all ( Table 4).

**Table 4.** Distribution of deaths by activity before drowning for the period 2013–2015.

Activity	Number (%)
Bathing	222 (14.7%)
Swimming	269 (17.9%)
Crossing river	244 (16.3%)
Accidently falling	211 (14.0%)
Fishing/boating	110 (7.3%)
Suicide	109 (7.2%)
Playing in the water source	101 (6.7%)
Falling from bridge	45 (2.9%)
Water-filled pits/pond/dam	15 (1.0%)
Others ^¶^	181 (12.0%)
**Total**	**1507 (100%)**

¶Others were the cases where activity before drowning were not clearly mentioned

Data file containing drowning data gathered from police records Click here for additional data file. Copyright: © 2018 Sedain B and Pant PR 2018 Data associated with the article are available under the terms of the Creative Commons Zero "No rights reserved" data waiver (CC0 1.0 Public domain dedication).

## Discussions

A systematic review on fatal river drownings showed that river drowning deaths are an issue in many regions and countries around the world ( Peden *et al*., 2016; Rahman *et al*., 2009). Similarly, epidemiological study conducted in Australia elucidated natural water bodies account for large proportion of drowning deaths ( Peden *et al*., 2016). However, there has been limited research that has explored drowning in locations other than beaches and swimming pools. Natural water bodies such as rivers, streams and lakes regularly account for large proportions of drowning deaths ( Peden *et al*., 2016; Rahman *et al*., 2017). Drowning, along with other injuries is neglected in Nepal. Most of the drowning occur in the natural water bodies such as rivers, canals, streams, lakes. In the *plains*, the number of drowning deaths were high although it was proportional to the population size. Nature of water bodies and landscape are different in three (Mountain, Hill and Terai) geographical regions, same as that the risk factors vary by geographical regions. Similarly, more than half of the drowning deaths occur in Hilly and Mountain regions.

A study by Pant and colleagues ( Pant *et al*., 2011) has a number of limitations; even though that demonstrated a similar type of results reported in this paper. Nepal is mountainous country, rivers are the major natural water body and rivers accounted for the largest number of fatal drownings (80%) for the period between 2013 and 2015. This was unlike other studies from LMICs ( Hyder *et al*., 2009; Rahman *et al*., 2009; Soko, 2012) where most of the drownings occurred in wells, water storage facilities or reservoirs, ditches and drains.

The monsoon season in Nepal takes place during the months of June–September when 80% of the yearly rainfall occurs throughout the country. During the months of April – June (summer), it is very warm during the day and more people use rivers, streams or other water bodies for swimming and bathing. Drownings occur while performing these activities. However, it was found drowning deaths occur throughout the year.

In the absence of household pipe water supplies and bridges, people are forced to be in contact with these unprotected water bodies every day. Data revealed that drowning often occurs as a result of the activities associated with daily life i.e. bathing, crossing river and fishing in the river. Surprisingly, no cases were reported to have drowned while fetching water which implies the need for more in-depth research.

Drowning varies greatly with age ( Pant *et al*., 2011; Quan & Cummings, 2003). This study also revealed that drowning is more common in children rather than older age groups. A similar finding has also been highlighted in several global ( World Health Organization, 2014), regional ( Quan & Cummings, 2003; World Health Organization, 2014) and country level studies ( Pant *et al*., 2011; Rahman *et al*., 2017). Drowning due to accidental falls mostly occurred in 0–4 year old children. These findings were similar to the study by Royal Life Saving Society in Australia ( RLSSA, 2016). This study also found that drowning claimed more lives of children below 18 years than adults.

Similarly, the proportion of drowning deaths were higher among males. Roughly three times as many deaths were reported in male versus female, which is similar to the findings of WHO’s Global Report on Drowning ( Rahman *et al*., 2017; Soko, 2012; World Health Organization, 2014; World Health Organization, 2014). A study on alcohol and its contributory role in fatal drowning in Australian rivers found that the alcohol consumption among male increased fatal drowning ( Peden *et al.*, 2017). The higher male drowning might be due to increased exposure to water and riskier behaviour. Findings show that female drowning was common in early ages (less than 9 years) and for the male it was high during the age 10–19 years. Overall, males of all ages outnumbered females for drowning.

These patterns of drowning in terms of age, gender, places of drowning, activity before drowning and seasonal patterns are consistent with findings of studies elsewhere ( IHME, 2016; Pant *et al.*, 2011; Peden *et al.*, 2016). Further study is needed to identify the tendency to exposure and age-related drowning by activity before drowning.

## Strengths

The strength of the study is that nationally recorded information from the Nepal Police was obtained. To our knowledge, this is the first study that attempted to study about drowning throughout the country which is able to present the patterns of drowning by district, age, gender and place of occurrence.

## Limitations

Information was limited to those drowning cases reported within 24 hours of occurrence through the daily incident reporting system of Nepal Police. This information doesn't take into account those who have gone missing into water. So, this study underreports drowning in Nepal. There is a lack of information on distance from home, time of the event, person accompanying the victims, and intent and influence of substances or illness responsible for the drowning incidents, which can be very helpful for designing drowning prevention interventions.

The data collected by the police was not be for the purpose of drowning prevention rather was for a forensic investigation or criminality. Some of the reported variables were incomplete (i.e. age of the person, location of drowning etc.). The data used in this manuscript has very limited information for every component of: a) victim information, b) scene information, c) any emergency medical services provided, and d) any Hospital care received by the victim.

## Conclusion

The findings of this study suggest that drowning occurs in many parts of Nepal and not necessarily only in the plains; and children are highly vulnerable to drowning. Rivers were the most common place of drowning in Nepal and the rate of drowning increased in rainy season. There is a need for better understanding of people's contact with rivers by gender and age to inform prevention. 

## Data availability

The data referenced by this article are under copyright with the following copyright statement: Copyright: © 2018 Sedain B and Pant PR

Data associated with the article are available under the terms of the Creative Commons Zero "No rights reserved" data waiver (CC0 1.0 Public domain dedication). 

Dataset 1: Data file containing drowning data gathered from police records 10.5256/f1000research.14563.d202804 ( Sedain & Pant, 2018)
