# Supplementary material for: Status of drowning in Nepal: A study of central police data
Source: F1000Res. 2018 Jul 30;7:576. Originally published 2018 May 14. [Version 2] doi: 10.12688/f1000research.14563.2 (PMC6113885; doi:10.12688/f1000research.14563.2)
Supplement: Peer review history

# Peer review: Referee response for version 2

**DOI:** 10.5256/f1000research.16953.r36594

**Published:** 2018-08-28

**Authors:** David Meddings

**Affiliations:** 1 Department for Management of Noncommunicable Diseases, Disability, Violence and Injury Prevention (NVI) , World Health Organization (WHO), Geneva, Switzerland

The revised version is improved significantly and I am fine to approve it now without reservations.

I have read this submission. I believe that I have an appropriate level of expertise to confirm that it is of an acceptable scientific standard.

# Peer review: Referee response for version 2

**DOI:** 10.5256/f1000research.16953.r36593

**Published:** 2018-08-13

**Authors:** Amy E. Peden

**Affiliations:** 1 Royal Life Saving Society – Australia, Sydney, NSW, Australia

Thank you for the chance to review the revised article. The authors have addressed my concerns sufficiently. I congratulate them on this important work on adding to the scant body of the literature on drowning in Nepal.

I have read this submission. I believe that I have an appropriate level of expertise to confirm that it is of an acceptable scientific standard.

# Peer review: Referee response for version 1

**DOI:** 10.5256/f1000research.15850.r34006

**Published:** 2018-05-16

**Authors:** David Meddings

**Affiliations:** 1 Department for Management of Noncommunicable Diseases, Disability, Violence and Injury Prevention (NVI) , World Health Organization (WHO), Geneva, Switzerland

Thank you for the opportunity to review this paper.

**General comments**

1. The paper is an important and useful contribution, as there is very little in the peer-reviewed literature about drowning in Nepal.
2. The authors should go into more detail about the police data source they used - particularly whether there are estimates of the number of deaths that occur but which are not reported to this source, whether there are biases etc.
3. The presentation of results seems somewhat haphazard - the authors could benefit from taking one step back from their data and deciding what are the 3-5 main findings we have come across here that are important and deserve to be amplified and framed clearly for the reader. 
4. There is little in the way of serious follow up discussion on some aspects that would seem to invite more critical thinking - the disparity between this study's findings and GBD estimates for example. 
5. There are numerous spelling and grammatical errors in the text, so this should be edited prior to publication.

**Specific comments**

1. Results section - drop "the open water" - which could be confused as meaning waters far from any coastline - and replace with either natural water bodies or rivers.
2. Proportion of males is cited as being higher than females - it is simple enough to give this as "roughly 3 times as many deaths were reported in males versus females", or words to that effect. It communicates with greater precision one of the findings of interest.
3. I take the seasonality pattern but believe there is somewhat muddled writing about the effect of seasons here. Clearly there are some drownings that occur year round, but the authors should be clear on drawing attention to the surge during the monsoon season as this has implications for prevention programmes.
4. The references appear incomplete - for example, the Global Report on Drowning from the WHO is referenced within the body of the text but does not appear within the references, although a WHO regional report does.

I have read this submission. I believe that I have an appropriate level of expertise to confirm that it is of an acceptable scientific standard, however I have significant reservations, as outlined above.

# Peer review: Referee response for version 1

**DOI:** 10.5256/f1000research.15850.r34009

**Published:** 2018-05-15

**Authors:** Amy E. Peden

**Affiliations:** 1 Royal Life Saving Society – Australia, Sydney, NSW, Australia

Thank you for the opportunity to review the article entitled “Status of drowning in Nepal: A study of central police data”. This is a well-written study detailing drowning in a country with little previous published research, using a novel data source being police data.

 I have some general comments for the authors that I hope they find useful.

 For someone unfamiliar with the geography of Nepal, I suggest adding a map as an additional figure that depicts the regions in Table 1 (Terai, Plains and Hills) geographically.

 You need to clarify somewhere in the methods (and abstract) that this is all drowning, both intentional and unintentional.

 I also have some specific comments for the authors as follows:

- Abstract - Methods – can you specify the dates of the study – e.g. 1 January 2013 and 31 December 2015
- Abstract – Methods – can you confirm what places means? Its not aquatic locations so is it geographical location within Nepal?
- Abstract – Methods – suggest replacing ‘done’ with ‘undertaken’ or ‘conducted’
- Main manuscript – Introduction – this study needs a clearer aim – the sentence that currently ends the introduction may be about the study referenced before. A clearer aim e.g. this study aims to …. Using police report as the main data source, with media reports as a secondary source of data..
- Methods – how many don’t report to police? Add to limitations.
- Methods – again confirm what place of drowning means if not type of body of water
- Ethics – usually even though the participants are deceased, this research would be considered as including human participants and ethics would be required. Is it possible to get ethics approval retrospectively? It is definitely helpful that you specify that the data received was de-identified.
- Results – can a definition for the term ‘open water’ as per the first paragraph of the results be added to the methods?
- Discussion – ‘systemic’ should be ‘systematic’.
- Discussion – please also cite dedicated epidemiological study of rivers that have been conducted in Australia ^1^. These further support the statement that natural water bodies such as rivers regularly account for large proportions of drowning deaths.
- Discussion – when discussing riskier behaviour in males – you might want to note the involvement of alcohol (see Peden et al. (2017) ^2^). I’m not sure whether alcohol is a big issue in Nepal, and I note there was no toxicological data from police supporting or disproving alcohol involvement but it might be worth mentioning as an additional area of research for drowning in Nepal, especially among males.
- Strengths - By using police records, you also avoid issues with traditional ICD drowning codes (W65-74) not capturing all drownings (see Peden et al. (2017) ^3^). This is a strength of the study.
- Limitations – are there ever cases of people not reporting a drowning at all or not reporting it within 24 hours? If so this study likely underreports drowning in Nepal and this would be worth mentioning.

I have read this submission. I believe that I have an appropriate level of expertise to confirm that it is of an acceptable scientific standard, however I have significant reservations, as outlined above.

## References

[1] PedenAEFranklinRCLeggatPA: The Hidden Tragedy of Rivers: A Decade of Unintentional Fatal Drowning in Australia. *PLoS One*.2016;11(8) : 10.1371/journal.pone.0160709 e0160709 10.1371/journal.pone.0160709 27517313PMC4982636

[2] PedenAEFranklinRCLeggatPA: Alcohol and its contributory role in fatal drowning in Australian rivers, 2002-2012. *Accid Anal Prev*.2017;98: 10.1016/j.aap.2016.10.009 259-265 10.1016/j.aap.2016.10.009 27771578

[3] PedenAFranklinRMahonyAScarrJBarnsleyP: Using a retrospective cross-sectional study to analyse unintentional fatal drowning in Australia: ICD-10 coding-based methodologies verses actual deaths. *BMJ Open*.2017;7(12) : 10.1136/bmjopen-2017-019407 10.1136/bmjopen-2017-019407 PMC577831929273670

# Response

**Published:** 2018-07-11

**Authors:** Bhagabati Sedain

**Affiliations:** Tribhuvan University, Nepal

For someone unfamiliar with the geography of Nepal, I suggest adding a map as an additional figure that depicts the regions in Table 1 (Terai, Plains and Hills) geographically.

**Author's** **response**:  Thank you! We found your comment extremely helpful and have revised accordingly.

 You need to clarify somewhere in the methods (and abstract) that this is all drowning, both intentional and unintentional.

**Author's Response:** Both the intentional and unintentional drowning deaths were analysed in this study. So, in this revised paper, it is clearly mentioned that the study included both the intentional and unintentional drowning deaths.

 I also have some specific comments for the authors as follows:

 Abstract - Methods – can you specify the dates of the study – e.g. 1 January 2013 and 31 December 2015

**Author's Response**: We also realised it is better to mention the study date in such way and have revised accordingly.

 Abstract – Methods – can you confirm what places means? Its not aquatic locations so is it geographical location within Nepal?

**Author's Response**: In the study, the places means the geographical locations i.e. Mountain, Hill and Terai. Instead of places, the term geographical location is used in the revised paper.

 Abstract – Methods – suggest replacing ‘done’ with ‘undertaken’ or ‘conducted’

**Author's Response**: We really appreciated your comments and since terminology would be more accurate and precise, we have taken your advice.

 Main manuscript – Introduction – this study needs a clearer aim – the sentence that currently ends the introduction may be about the study referenced before. A clearer aim e.g. this study aims to …. Using police report as the main data source, with media reports as a secondary source of data..

**Author's Response**: The study used police report as the main source of data. Objectives of the study was mentioned in the abstract. We also noticed that the objective of the study was missing in the introduction section of the main text. At the end of the introduction of the main text, the objective of the study is stated in the revised article.

 Methods – how many don’t report to police? Add to limitations.

**Author's Response**:  Review of literature showed that larger number of unnatural deaths were not reported in the police. As Nepal is a less developed country, there is the high probability of people not reporting the unnatural deaths to the police. So, it is challenging to discuss on the missing cases. In case of drowning deaths, Nepal Police’s record is the only one source information.

 Methods – again confirm what place of drowning means if not type of body of water

**Author's Response**: The word drowning place was mistakenly written in the main text so it is deleted.

 Ethics – usually even though the participants are deceased, this research would be considered as including human participants and ethics would be required. Is it possible to get ethics approval retrospectively? It is definitely helpful that you specify that the data received was de-identified.

**Author's Response**: Ethical approval was obtained from University Review Committee. We used secondary data which was completely de-identified right from Data Extraction which was done on the Police Office premises. No identifiable data for the deceased person was transferred by any means.

 Results – can a definition for the term ‘open water’ as per the first paragraph of the results be added to the methods?

**Author's Response**: Thank you for this excellent observation. Both of the reviewers commented on this word ‘open water’. So the word replace with natural water bodies.

 Discussion – ‘systemic’ should be ‘systematic’.

**Author's Response**: It was a typo in the manuscript and changed accordingly.

 Discussion – please also cite dedicated epidemiological study of rivers that have been conducted in Australia1. These further support the statement that natural water bodies such as rivers regularly account for large proportions of drowning deaths.

**Author's Response**: We agree that it will be better if it was included in the discussion. We have added the suggested reference in the discussion section.

 Discussion – when discussing riskier behaviour in males – you might want to note the involvement of alcohol (see Peden et al. (2017)2). I’m not sure whether alcohol is a big issue in Nepal, and I note there was no toxicological data from police supporting or disproving alcohol involvement but it might be worth mentioning as an additional area of research for drowning in Nepal, especially among males.

**Author's Response:** In the police record, the use alcohol use was not clearly mentioned. However, there is the high prevalence of drinking behaviour among the male. So we are grateful to know about this article and it was realised that the adding of the suggested reference would validate our result. So the suggested reference has been included in the revised manuscript.

 Limitations – are there ever cases of people not reporting a drowning at all or not reporting it within 24 hours? If so this study likely underreports drowning in Nepal and this would be worth mentioning.

**Author's Response** **:** . There is high possibility of not reporting all deaths within 24 hrs. The information recorded in the Nepal Police through daily incidence reporting system was analysed in this study. It is likely to observe underreporting of the drowning deaths. In the revised manuscript this issue is clearly mentioned in the limitation section.
